# Bilateral Optic Atrophy in a Young Patient With Chronic Anaemia Secondary to End-Stage Renal Disease

**DOI:** 10.7759/cureus.13969

**Published:** 2021-03-18

**Authors:** Nor Hasnida AB Gani, Mohtar Ibrahim, Wan-Hazabbah Wan Hitam, Nurul Ain Masnon, Amirah Hassan

**Affiliations:** 1 Department of Ophthalmology, Department of Ophthalmology and Visual Science, School of Medical Sciences, Universiti Sains Malaysia/Hospital Universiti Sains Malaysia, Kubang Kerian, MYS; 2 Ophthalmology, Hospital Serdang, Kajang, MYS

**Keywords:** optic atrophy, anaemia, end-stage renal failure, young

## Abstract

End-stage renal disease (ESRD) is associated with a number of serious complications, including increased cardiovascular disease, anaemia and metabolic bone disease. Optic atrophy secondary to chronic anaemia in ESRD is rare. We report a case of bilateral optic atrophy in a young patient with chronic anaemia secondary to ESRD. A 23-year-old lady with ESRD, presented with progressive blurring of vision in her left eye for a period of six months. Visual acuity in the left eye was counting finger and the right eye was 6/6. Left optic nerve functions were significantly reduced. Bilateral anterior segments and intraocular pressure were normal. Funduscopy showed bilateral pale disc with arteriolar attenuation. The infective, autoimmune and demyelinating screening were negative. Serial full blood count indicated low haemoglobin and haematocrit value. The full blood picture revealed normocytic normochromic anaemia. Neuroimaging was normal. The patient was diagnosed as having bilateral optic atrophy secondary to chronic anaemia due to ESRD. Chronic anaemia is a potential cause of optic atrophy in a young patient with chronic disease. Management of anaemia in such cases is crucial to prevent irreversible complications including optic atrophy and blindness.

## Introduction

End-stage renal disease (ESRD) has emerged as a global public health problem. The Asian population has the highest prevalence of ESRD in the world. In countries like China, the Philippines and Malaysia, the annual growth of ESRD exceeds more than 10% due to aging population and underlying diabetes and hypertension [1]. Demographically, Asia’s ESRD population has two distinct features: a relatively younger age compared to developed countries (at least two decades younger) and a high prevalence (17%-20%) of chronic kidney disease (CKD) of uncertain etiology [1]. ESRD is associated with a number of serious complications, including increased incidence of cardiovascular disease, hyperlipidemia, anaemia and metabolic bone disease. ESRD patients should receive optimal treatment to reduce their morbidity and mortality. This case report demonstrates the importance of anaemia management in ESRD patients, in order to avoid irreversible sequelae such as bilateral optic atrophy in young patients.

## Case presentation

A 23-year-old female came with progressive left eye blurring of vision for six months duration. She denied any eye pain, headache, nausea or vomiting. There was no limb weakness, unsteady gait and bowel or bladder dysfunction. There was also no facial or body rashes, joint pain, mouth ulcer or hair loss. No family history of blindness or neurological problem. She was diagnosed with ESRD two years ago after she had community-acquired pneumonia (CAP) complicated with oliguric acute kidney injury and severe metabolic acidosis. The kidney function deteriorated further and she required continuous ambulatory peritoneal dialysis (CAPD) four times daily.

**Figure 1:**
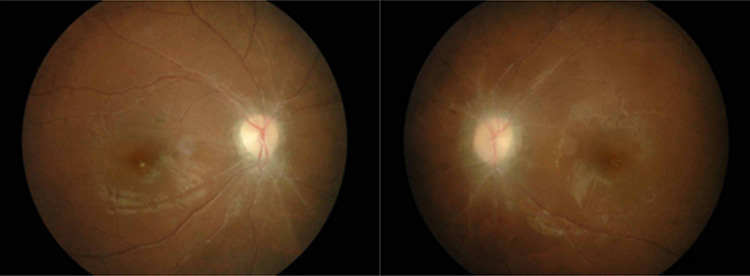
Bilateral optic disc atrophy with attenuated arteriolar and dilated veins.

**Figure 2:**
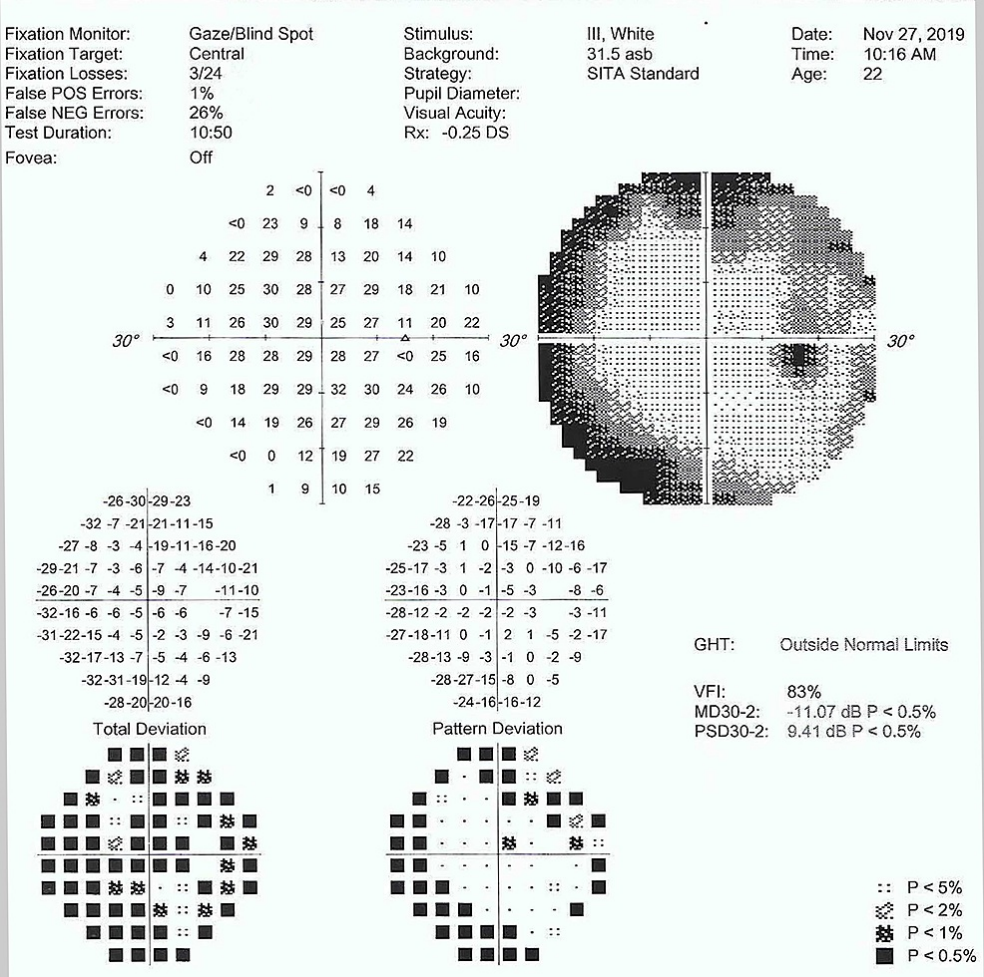
Humphrey visual field test in 2019.

On examination, her visual acuity was 6/6 on the right eye and counting finger on the left eye. The optic nerve function was reduced on the left eye. Both eyes had full range of extraocular movement, normal anterior segments and intraocular pressure. Funduscopy revealed bilateral optic atrophy cup disc ratio of 0.3, arteriolar attenuation and slight venous tortuosity (Figure 1). Humprey visual field test for her right eye showed restriction of visual field (Figure 2) while the left eye visual field could not be plotted due to poor vision. Blood pressure and heart rate were in higher side. Blood glucose was normal.

Serial full blood count for two years indicated persistence low haemoglobin (6.8-7.5 g/dL) and haematocrit value (21.3%-22.3%). The full blood picture showed normocytic normochromic anaemia. Her average serum iron was 4.7-13.4 µmol/L, with low total iron binding capacity 25.6-33.1 µmol/L and unbound iron binding capacity range was 12.7-28.4 µmol/L. The serum creatinine was high (1202-1683 µmol/L). Thyroid function test was within the normal range. Plasma intact parathyroid hormone (iPTH) level was elevated at 460 pg/mL. Serum total Calcium was in lower side (2.17-1.78 mmol/L). Serum phosphate inorganic was in higher level (2.31-3.49 mmol/l). The infective and autoimmune screening were normal. Serum aquaporin-4 receptor antibody and myelin-oligodendrocyte glycoprotein (MOG) antibody were negative. Magnetic resonance imaging (MRI) brain and orbit revealed normal brain parenchyma with no space occupying lesion hydrocephalus or periventricular plaques. We diagnosed this patient with bilateral optic disc atrophy secondary to chronic anaemia due to ESRD.

The patient was given subcutaneous erythropoiesis-stimulating agent 4000IU three times per week for anaemia treatment since one month on dialysis. She was also given tablet ferrous fumarate, vitamin B complex, folic acid 5 mg, calcium carbonate and potassium chloride. She was emphasised on compliance and able to tolerate the oral medications. Her haemoglobin improved to 10.2 g/dL after 5 months. Despite of increasing trend of the haemoglobin level, there was no improvement of the visual functions. Serial follow-up demonstrated the visual acuity remains the same and the visual field was noted to further worsen over the right eye.

## Discussion

Anaemia is particularly prevalent among patients with CKD. It increases in frequency and severity in the more advanced stages of CKD. The prevalence of anaemia is double in people with CKD (15.4%) compared to general population (7.6%) [2]. It increases with the stage of CKD, from 8.4% at stage 1 to 53.4% at stage 5 or ESRD [2]. The anaemia in ESRD patients raise the morbidity and mortality, particularly from cardiovascular complications such as angina, left ventricular hypertrophy and heart failure [3]. Anemia in ESRD also led to symptoms of fatigue, dizziness, cognitive impairment and reduced quality of life.

Anaemia in ESRD is typically normocytic, normochromic and hypoproliferative [4]. While anaemia in ESRD can result from multiple mechanisms (iron, folate or vitamin B12 deficiency, gastrointestinal bleeding, severe hyperparathyroidism, systemic inflammation and shortened red blood cell life span), a decrease in erythropoietin synthesis is the most important and specific etiology causing ESRD-related anaemia [5-7]. Erythropoietin is a glycoprotein secreted by the kidney interstitial fibroblasts and is essential for the growth and differentiation of red blood cells in the bone marrow [5]. It maintains the homeostasis in the red blood cell supply to achieve adequate tissue oxygen delivery. Decrease in erythropoietin synthesis could resulted from tubulointerstitial fibrosis in ESRD, iron loss due to dialysis and high PTH level that downregulate the erythropoiesis [5,7].

Anaemia is a known cause of optic atrophy in ESRD patients, with several cases has been reported previously in the literature which was severe hypotension, atherosclerosis and hyperuremic optic atrophy (Table 1) [8-10]. Compared to the reported cases, our case was among the youngest and had the shortest duration of ESRD before she developed optic atrophy. Severe anaemia may lead to retinopathy and optic neuropathy. It has been postulated that persistently low haematocrit could affect the blood’s oxygen-carrying capacity, therefore causing ischaemia to the nerves [8].

**Table 1:** Reported cases of optic atrophy in end-stage renal disease. VA = visual acuity, BE = both eyes, RE = right eye, LE = left eye, CF = counting finger, NPL = non perception of light, ESRD = end-stage renal disease, CAPD = continuous ambulatory peritoneal dialysis.

Journal (year)	Age	Associated medical problem	Duration of ESRD (year)		Method of dialysis	Laterality	VA on presentation	VA post-treatment
Haider et al. (1993) [8]								
Case1	53	DM type 1 Polycystic kidneys Anaemia		25	Haemodialysis	Unilateral LE	LE 6/6	LE 6/6 with altitudinal defect
Case 2	42	Bilateral small kidneys Anaemia		5	Dialysis	Unilateral LE	LE 6/9	LE 6/9
Case 3	77	Hypertension Congestive cardiac failure Ischemic Heart Disease Anaemia		20	CAPD	Bilateral	RE 6/60 LE CF	Not mention
Case 4	32	DM type 1 Ischemic Heart Disease Anaemia		Not mention	Haemodialysis (intermittent)	Bilateral	RE CF	RE 6/18
Basri and Shaheen (2002) [9]								
Case 1	40	Hypertension	16		Haemodialysis	Bilateral	BE NPL	BE NPL
Nieto and Zapata (2010) [10]								
Case 1	26	Urethral diverticle Hypotension Chronic anaemia	20		Haemodialysis	Unilateral LE	LE 6/60	LE NPL
Case 2	56	Unknown cause	Not mention		CAPD	Unilateral RE	RE inferior altitudinal defect	RE CF persistent altitudinal defect
Our case (2021)								
Case 1	23	Glomerulonephritis Hypertension	2		CAPD	Unilateral LE	RE 6/6 LE 6/36	RE 6/9 LE CF

Lack of iron is thought to be associated with hypomyelination of the nerve fibres as iron is a vital element for myelin production. It becomes a risk for optic nerve which its major components are myelinated fibres [11]. The deficiency of vitamin B12 also can cause optic atrophy [12].

On the other hand, the uremic condition is neurotoxic to the optic nerve head. However, the pathogenesis of uremic optic neuropathy is poorly understood and has been postulated due to dialyzable toxic metabolites [13]. Hypothetically, a neurotoxic type of optic neuropathy occurs if the urea level is higher than 35.7mmol/l [8]. Nevertheless in our patient, her average urea on dialysis was between 13.6-21.5mmol/l. The level might not high enough to a cause toxic effect to the optic nerve for our patient.

In a young patient with bilateral optic atrophy, the differential diagnosis includes long-standing raised high intracranial pressure, infective, demyelinating, autoimmune, toxic, nutritional and hereditary causes. All these potential causes have been excluded in our patient. MRI imaging, cerebrospinal fluid analysis and other blood investigation results were normal. The persistently low hemoglobin with normocytic normochromic features together with elevated serum iPTH and phosphate that we found in our patient were consistent with ESRD-related anemia.

Anaemia in dialysed patients should be corrected until achieved the haemoglobin level of 10-11.5g/dl [14]. Methods of corrections include intravenous or subcutaneous erythropoiesis-stimulating agent, intake of ferrous fumarate, vitamin B complex and folic acid. Our patient received subcutaneous Epoetin beta which is the recombinant form of erythropoietin. These drugs act on the erythro progenitor cells. It increases the reticulocytes counts, haemoglobin levels and hematocrit in dose-proportional manner. It could increase the haemoglobin levels and hematocrit around 15% to 54% and 17-60% respectively within eight weeks to 12 months duration [15]. Supplements such as ferrous fumarate, vitamin B complex and folic acid are required to replenish the depletion during haemodialysis [5].

The persistent poor visual functions in our case could be due to long-standing anaemia and the delay in achieving the optimum level of haemoglobin. Nevertheless, the prognosis patient with anaemic optic neuropathy is guarded. A good prognosis depends on the early detection of the causes and the effectiveness of the treatment. Multidisciplinary follow-up is necessary for ESRD patients for optimal therapy and to detect early complications of the disease. Optic atrophy is irreversible. Low vision aid and visual rehabilitation may help those patients with late-stage optic atrophy in ESRD-related anemia.

## Conclusions

Achieving optimum haemoglobin level is crucial in ESRD patients. Chronic severe anaemia will cause devastating complications such as optic atrophy which is lead to irreversible blindness. Patients with ESRD on regular dialysis with persistent anaemia need to be co-managed by nephrologist, physician, haematologist and ophthalmologist. Early eye assessment is necessary. Even in young ESRD patients, persistent anaemia is a risk for them to get optic atrophy. Improving our understanding on the multifaceted process of anemia in ESRD means better management and outcome in our practice.
